# STYX/FBXW7 axis participates in the development of endometrial cancer cell via Notch–mTOR signaling pathway

**DOI:** 10.1042/BSR20200057

**Published:** 2020-04-17

**Authors:** Liheng Liu, Haili Jiang, Xiaoxin Wang, Xin Wang, Liying Zou

**Affiliations:** Department of Obstetrics, Beijing Obstetrics and Gynecology Hospital, Capital Medical University, Beijing 100026, China

**Keywords:** apoptosis, endometrial cancer, FBXW7, proliferation, STYX

## Abstract

Endometrial cancer (EC) is the most common gynecologic malignancy in world. It has been reported that the mutation rate of FBXW7 is frequent in EC, but the specific functions of FBXW7 remain unknown in EC. In the present study, we revealed the role and mechanism of FBXW7 in EC cells. Compared with adjacent nontumor tissues, the FBXW7 expression level was lower in EC tissues. However, the level of STYX was in contrast with the expression of FBXW7 in EC tissues. And STYX interacted with FBXW7 and then down-regulated its expression level in EC. Over-expression of FBXW7 inhibited cell proliferation and facilitated apoptosis in EC cells, whereas silencing FBXW7 acted an opposite effect on EC cells. And the process of FBXW7 participated the proliferation and apoptosis in EC was regulated by STYX. FBXW7 suppressed the expression of Notch pathway related protein, and further inhibited the phosphorylation of mTOR. In addition, we also found that mTOR activitor (MHY1485) and Notch activator (Jagged-1) reversed the effect of over-expressing FBXW7 on cell proliferation and cell apoptosis. And Notch inhibitor (DAPT) counteracted the impact of over-expressing STYX on cell proliferation and cell apoptosis. Collectively, the present study verified that STYX inhibited the expression level of FBXW7 in EC, and then promoted cell proliferation but suppressed apoptosis through Notch–mTOR signaling pathway, which promoted carcinogenesis and progression of EC.

## Introduction

Endometrial cancer (EC) originates from the lining of the uterus [1], which is the most prevalent malignant tumors and one of the most common reasons of death of women’s cancers [2–4]. Recently, the incidence of EC has been rising annually and the disease onset has gradually become younger [5]. Despite most EC patients can be diagnosed and treated in the early stage of EC, approximately 15% of EC patients are diagnosed with advanced or occult metastases, resulting in poor outcome and even high mortality [6–8]. Generally speaking, accumulation of multiple genetic abnormalities (which is likely to inactivate tumor-suppressor genes and activate oncogenes) lead to the arise of EC [9]. Nevertheless, the molecular pathogenesis of EC has not yet been fully elucidated. Hence, it is pressing to research the underlying molecular mechanisms of EC, so as to provide new directions for its treatment.

Serine/threonine/tyrosine interacting protein (STYX), a protein of protein tyrosine phosphatases (PTPs) family, is a prototypical pseudophosphatase [10]. Several researches indicated that STYX has a diffused expression in multiple tissues; however, it is less to known about its biological function in cancers [11,12]. Reiterer et al. illustrated that STYX acted as a nuclear anchor to adjust the nucleo-cytoplasmic shuttling of ERK1/2 (extracellular signal-regulated kinases 1/2) [13]. Wishart et al. identified that STYX coupled with a testicular RNA-binding protein, indicating the underlying character of STYX in spermatogenesis [14]. He et al. study attested that STYX is connected with cell proliferation, migration, invasion and apoptosis in colorectal cancer cells [11]. A majority of study testified STYX acts as a latent oncogene, suggesting that STYX restrained cell apoptosis in colorectal and breast cancer by binding F-box and WD40 domain protein 7 (FBXW7, also called hCDC4, Fbw7) protein [11,15].

FBXW7 is a conserved F-box WD40 protein, which functions as a substrate recognition subunit of the SCF (SKP1/CUL1/F-box protein) E3 ubiquitin ligase [16]. Several studies have attested that FBXW7 plays tumor suppressor roles in human cancer [17,18]. A large amount of cancer-related mutations of FBXW7 have been verified in various cancers and loss of FBXW7 function leads to carcinogenesis [19]. Levine has identified that FBXW7 has 82% mutation rate in EC [1]. It is now well established that FBXW7 functions as a tumor suppressor that promotes apoptosis in human tumor cells [16]. For example, FBXW7 inhibits the proliferation and migration as well as the promotion of apoptosis of CRC cells and acts as a negative regulation in the pathogenesis of CRC [20–22]. And FBXW7 targets several proteins with critical roles in the hallmarks of cancer, such as cyclin E, Notch, mTOR, c-MYC, etc [17,23–25]. Based on these results, we speculate that FBXW7 plays a crucial role in the progression of EC.

The Notch is an evolutionally conserved signaling pathway that has been involved in multifarious processes, including determination of cell fate, regulation of cell proliferation, differentiation and cell death [26]. Notch signaling has been diffusely researched in various gynecologic cancers, such as cervical cancer, ovarian cancer and endometrial cancer [27]. Furthermore, Notch activator accelerates the expression and phosphorylation level of the mTOR protein and the activation of mTOR signaling [28]. mTOR plays a significant role in cell growth and survival, and has latterly been demonstrated in EC pathogenesis [29].

In the present study, we demonstrate that the function and mechanism of FBXW7 in EC. STYX and FBXW7 expression were measured both in EC tumor and normal tissues. FBXW7 suppressed cell proliferation and promoted cell apoptosis in EC, and our study found that STYX directly interacted with FBXW7 and then adjusted its function in EC cells. Furthermore, Notch and mTOR signaling pathways may be associated with FBXW7-mediated regulation of proliferation and apoptosis in EC cells. In general, it is identified that STYX/FBXW7 axis participates the development of EC via Notch–mTOR signaling pathway.

## Methods

### Patients and specimens

Human endometrial tissue samples were acquired from 20 women experiencing surgery during 2018–2019. The tissue samples were obtained from Beijing Obstetrics and Gynecology Hospital, Capital Medical University. Every tumor sample had a relative adjacent nontumor endometrial tissue as control. All of the tissue samples were snap frozen immediately in liquid nitrogen, and then stored it at -80°C until analysis. The whole patients hadn’t received any chemotherapy or radiotherapy before their surgery. This research was permissive by Ethics Committee of Beijing Obstetrics and Gynecology Hospital, Capital Medical University. And every patient afforded a written consent inform to us.

### Cell culture

HEC-1A, HEC-1B, Ishikawa, RL95-2 and AN-3CA cells were acquired from the Model Culture Collection (ATCC, Manassas, VA, U.S.A.). Mc5Coy’s5a medium was used in culturing HEC-1A cells. RPMI-1640 (Roswell Park Memorial Institute-1640) medium was used to culture Ishikawa cells. DMEM (Dulbecco’s Modified Eagle Medium) was applied to culture other cells. All medium contained 10% FBS (fetal bovine serum), 100 U/ml penicillin and 0.1 mg/ml streptomycin. Total cells were cultivated in a humidified condition at 37°C and 5% CO_2_. Reagents used in cell culture were bought from Gibco (Grand Island, NY, U.S.A.).

### Cell transfection

pcNDA3.1-FBXW7, FBXW7 shRNAs (shFBXW7) and STYX shRNAs (shSTYX) were acquired from Genechem Co. Ltd. (Shanghai, China). All cell lines were cultivated in six-well plates at a density of 5 × 10^5^ cells/well, and incubation overnight. Lipofectamine 2000 reagent (Invitrogen, Carlsbad, CA, U.S.A.) was used to transfect the vectors or miRNAs into incubated cells. And then incubated for another 48 h in a humid condition with 5% CO_2_ at 37°C.

### qRT-PCR

Total RNA was isolated from human endometrial tissue samples using TRIzol® Reagent Invitrogen 155 (Invitrogen, U.S.A.). PrimeScript RT Master Mix kit (Takara, Dalian, China) was used to reversely transcribed RNA samples. The expression level of mRNA was examined by using SYBR Premix Ex Taq™ (Takara Bio, Otsu, Japan). GAPDH was considered as the internal reference gene. 2^−ΔΔCq^ was used in representing the relative expression levels of gene.

### Western blot

Total tissue and cell were harvested and then lysed in RIPA buffer (50 mM Tris pH 7.5, 0.5% sodium deoxycholate, 150 mM NaCl, 10 mM NaF, 0.1% SDS, 1% Triton X-100) added protease inhibitors (Roche, Switzerland). The lysates were centrifuged at 12,000 ***g*** for 25 min at 4°C. Then, the concentration of protein was examined by the BCA Protein Assay kit (Genstar, China). Protein samples were separated by 10% SDS-PAGE after incubation at 95°C for 15 min in SDS sample buffer, and then transferred to PVDF membranes (Millipore, Boston, MA, U.S.A.). Next, the membranes were blocked with 5% (w/v) evaporated milk in TBST for 1 h at 25°C. The blocked membranes were put into TBST solution that contains primary antibodies (anti-FBXW7, anti-STYX and anti-GADPH) at 4°C overnight, and then washing five times with TBST solution. The PVDF membranes were incubated for 1 h at room temperature in IgG horseradish peroxidase secondary antibody (Sigma-Aldrich). After washing three times with TBST, they were imaged using StarSignal Plus Chemiluminescent Assay Kit (Genstar, China).

### Co-immunoprecipitation (Co-IP)

To acquire protein, all cells were lysed in RIPA buffer. Primary antibody (4 μg) was mixed with 1000 μg of total protein sample, and then incubated the mixture at 4°C for 8 h. Next, the protein A Sepharose beads (Santa Cruz, Texas, U.S.A.) were added to the antibody–protein mixture and incubated at 4°C for 1 h. The beads were centrifuged about 3 min at 800 ***g***. The 5× sample loading buffer was added into the Sepharose beads–antigen–antibody complexes and resuspended it at the same time. And then Western blot was used to examine the protein sample.

### MTT assay

Cell proliferation was measured by the MTT Cell Proliferation and Cytotoxicity Assay Kit (Beyotime, China).

### Apoptosis analysis

Apoptosis analysis was examined by Annexin V-FITC/PI Apoptosis Detection kit (KeyGen Biotech, Nanjing, China).

### Caspase 3 analysis

The activity of Caspase 3 protein was detected using Caspase 3 Activity Assay kit (Beyotime Biotechnology, Shanghai, China).

### Statistical analysis

All assays were conducted in triplicate. Statistical analysis was experiment with SPSS 22.0 software (SPSS, Chicago, IL, U.S.A.). The Student’s *t*-test was used to analyze the differences between two groups. When *P* values less than 0.05, differences were considered statistically significant.

## Results

### FBXW7 is down-regulated in endometrial cancer tissues, while STYX is up-regulated

**Figure 1:**
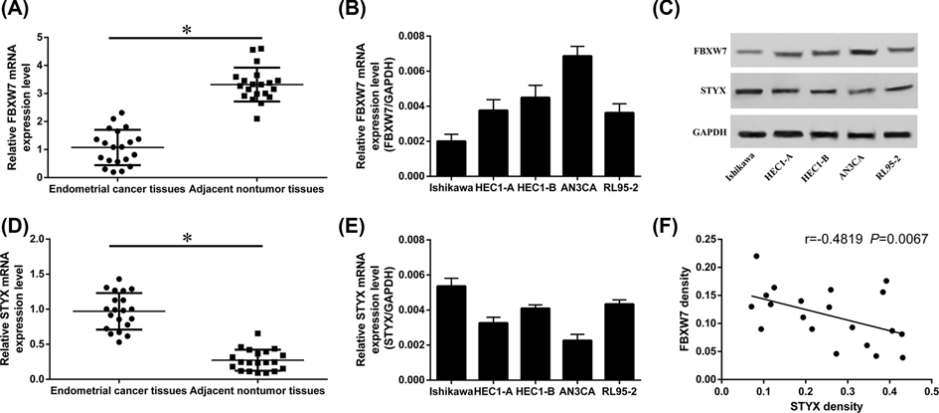
FBXW7 is down-regulated in endometrial cancer tissues, while STYX is up-regulated (**A–E**) Expression of FBXW7 and STYX in endometrial cancer tissues and cells are measured by qRT-PCR and Western blot. *P<0.05, compared with control. (**F**) FBXW7 and STYX correlated negatively in gastric cancer tissues, based on Pearson’s correlation curve.

We first determined the expression levels of FBXW7 and STYX in 20 cases of EC samples and normal endometrium samples, respectively. A lower FBXW7 expression and a higher expression of STYX were observed in human endometrial cancer tissues (Figure 1A,D). Spearman’s correlation analysis further proved that the expression of FBXW7 correlated negatively with STYX in endometrial cancer tissues. (Pearson *r* = −0.5855, *P* = 0.0067, Figure 1F). We also examined the expression level of FBXW7 and STYX in endometrial cancer cell lines (Figure 1B,C,E). We found that the expression level of FBXW7 was the lowest in Ishikawa and the highest in AN3CA (Figure 1B). However, the STYX expression was on contrary with FBXW7 expression in EC cells (Figure 1E).

### STYX interacted with FBXW7

**Figure 2:**
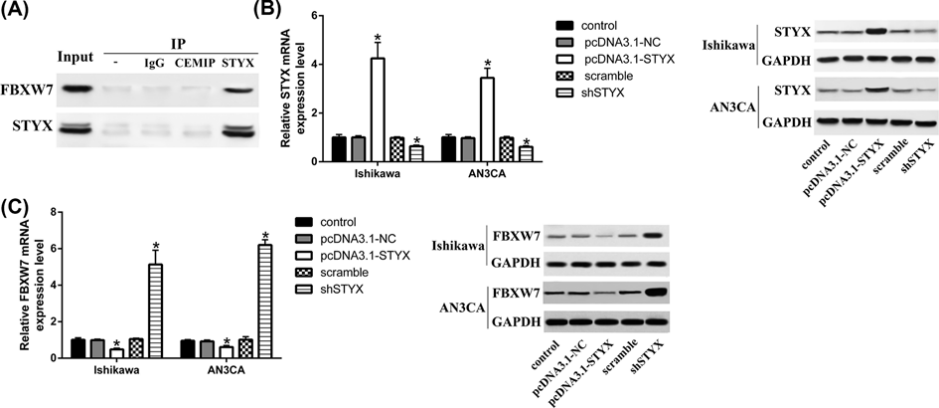
STYX interacted with FBXW7 (**A**) Interaction of STYX and FBXW7 is validated by co-IP. (**B** and **C**) STYX and FBXW7 expression in over-expressing and silencing STYX cell lines; *P<0.05, compared with control.

To certify the relationship between STYX and FBXW7, we carried out Co-IP assays first. The Co-IP results suggested that endogenous STYX interacted with FBXW7 in EC cells (Figure 2A). We next transfected shSTYX and pcDNA3.1-STYX into endometrial cancer cell line Ishikawa and AN3CA, respectively. The Western blot and qRT-PCR experiments validated that the expression level of STYX was up-regulated by pcDNA3.1-STYX and down-regulated by shSTYX in endometrial cancer cell (Figure 2B). Further, we found that FBXW7 was up-regulated after silencing STYX in EC cells, and down-regulated after over-expressing STYX in EC cells compared with control cells (Figure 2C).

### FBXW7 inhibition promotes the proliferation and suppresses the apoptosis of endometrial cancer

**Figure 3:**
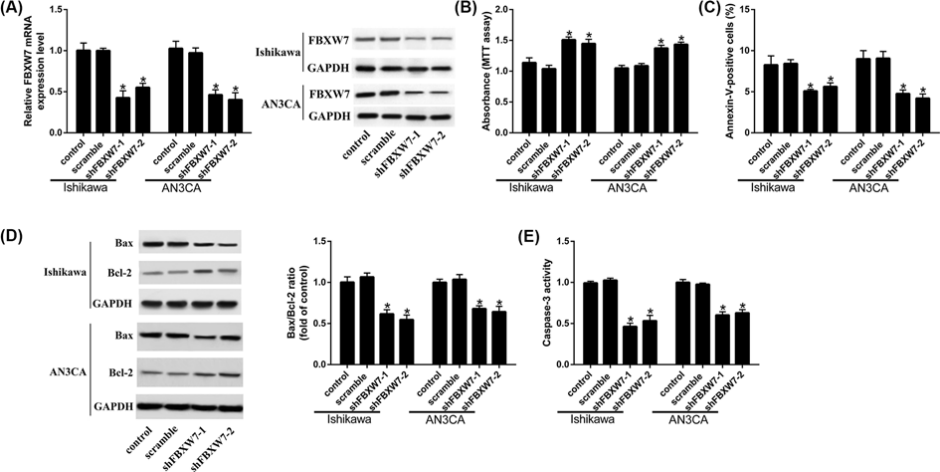
FBXW7 inhibition promotes the proliferation, but suppresses the apoptosis of endometrial cancer (**A**) FBXW7 expression after silencing FBXW7. (**B**) Cell proliferation is measured by MTT assay. (**C**) Cell apoptosis is measured by Annexin V-FITC/PI double stain. (**D**) Bax and Bcl-2 were measured by Western blot. (**E**) Caspase 3 activity is detected by Caspase 3 Activity Assay kit; *P<0.05, compared with control.

FBXW7-shRNAs were transfered into Ishikawa and AN3CA cells to knockdown FBXW7 (Figure 3A). Results implied that FBXW7 inhibition significantly increased the proliferation (Figure 3B) and reduced the apoptotic rate of EC cells (Figure 3C). We also measured the expression of Bax/Bcl-2 and the activity of Caspase-3 protein. The results indicated that the expression of Bax and the activity of Caspase-3 protein in two shFBXW7 groups were reduced when compared with the control group, while the level of Bcl-2 was increased. (Figure 3D,E).

### Over-expression of FBXW7 suppresses the proliferation, and promotes the apoptosis of endometrial cancer

**Figure 4:**
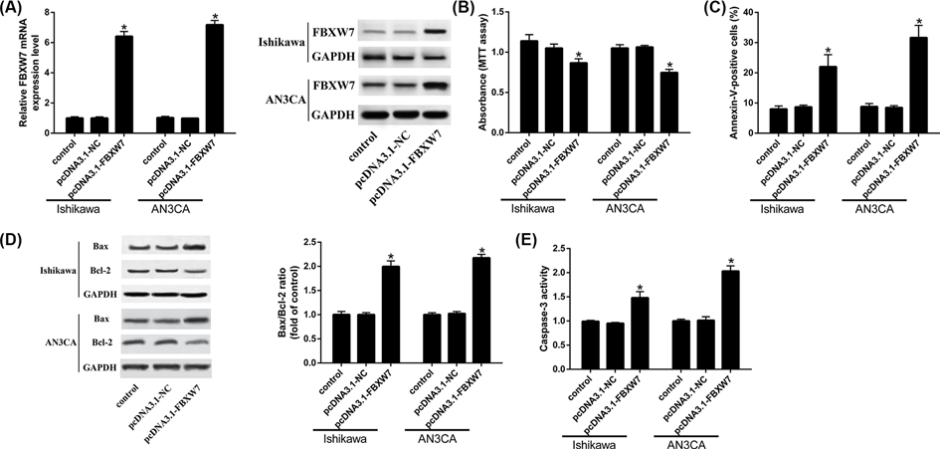
Over-expression of FBXW7 suppresses the proliferation, but promotes apoptosis of endometrial cancer (**A**) FBXW7 expression after over-expressing FBXW7. (**B**) Cell proliferation is measured by MTT assay. (**C**) Cell apoptosis is measured by Annexin V-FITC/PI double stain. (**D**) Bax and Bcl-2 were measured by Western blot. (**E**) Caspase 3 activity is detected by Caspase 3 Activity Assay kit; *P<0.05, compared with control.

We next transfected pcDNA3.1-FBXW7 into EC cells, and identified the level of FBXW7 was up-regulated in pcDNA3.1-FBXW7 transfected EC cells (Figure 4A). Over-expression of FBXW7 decreased cell proliferation of EC cells (Figure 4B). The apoptotic rate of EC cells was promoted after over-expression of FBXW7 (Figure 4C). The expression of Bax and Caspase-3 protein activity in the pcDNA3.1-FBXW7 group was higher than control group, while the level of Bcl-2 was lower. (Figure 4D,E).

### FBXW7 regulates the development of EC through Notch–mTOR pathway

**Figure 5:**
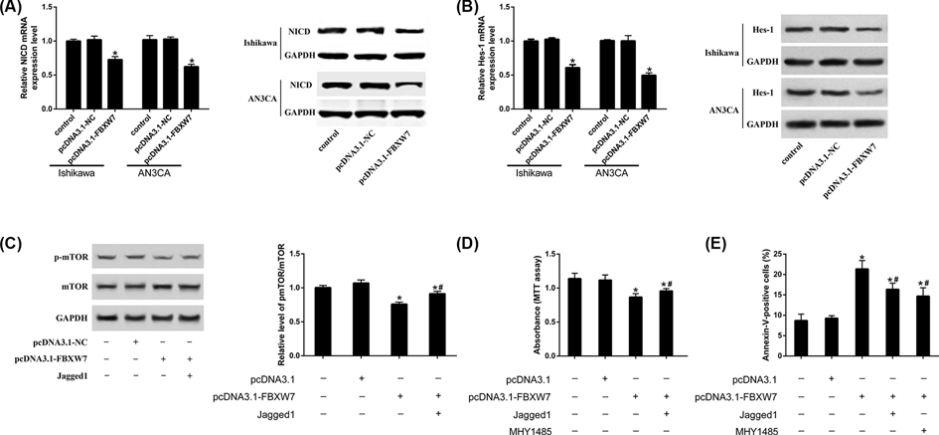
FBXW7 regulates the development of EC through Notch-mTOR pathway (**A** and **B**) NICD and Hes-1 expression are examined by qRT-PCR and Western blot. (**C**) The ratio of p-mTOR/mTOR. (**D**) Cell proliferation is measured by MTT assay. (**E**) Cell apoptosis is measured by Annexin V-FITC/PI double stain; *P<0.05, compared with control. ^#^*P*<0.05, compared with pcDNA3.1-FBXW7.

Several reseaches illustrated that FBXW7 plays critical roles in cancers by regulating Notch and mTOR [17,30]. We found that over-expression of FBXW7 resulted in a significant decrease in endogenous NICD level (Figure 5A). The expression of Hes-1 was also reduced after over-expressing FBXW7 (Figure 5B). Over-expressing FBXW7 decreases p-mTOR protein expression, while Jagged1 (the activator of Notch signaling) attenuates the effect of over-expressing FBXW7 on EC cells (Figure 5C). Over-expressing FBXW7 inhibited cell proliferation and suppressed apoptosis of EC cells (Figure 5D,E). The use of Jagged1 and MHY1485 (the activitor of mTOR signaling) partly rebounded the impact of over-expressing FBXW7 on cell proliferation and apoptosis (Figure 5D,E).

### STYX regulates cell proliferation and apoptosis by regulating FBXW7 in EC

**Figure 6:**
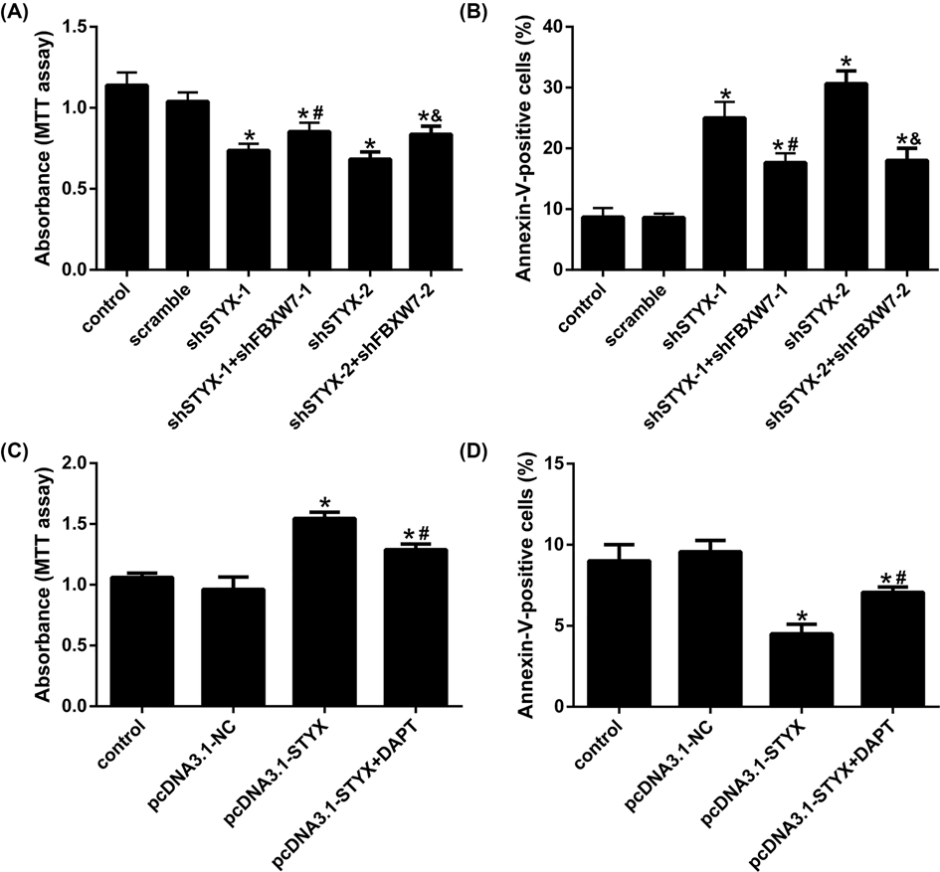
STYX regulates cell proliferation and apoptosis by regulating FBXW7 in EC (**A** and **C**) Cell proliferation is measured by MTT assay. (**B** and **D**) Cell apoptosis is measured by Annexin V-FITC/PI double stain; *P<0.05, compared with control. ^&^*P*<0.05 compared with sh-STYX-1 or sh-STYX-2. ^#^*P*<0.05, compared with pcDNA3.1-STYX.

**Figure 7:**
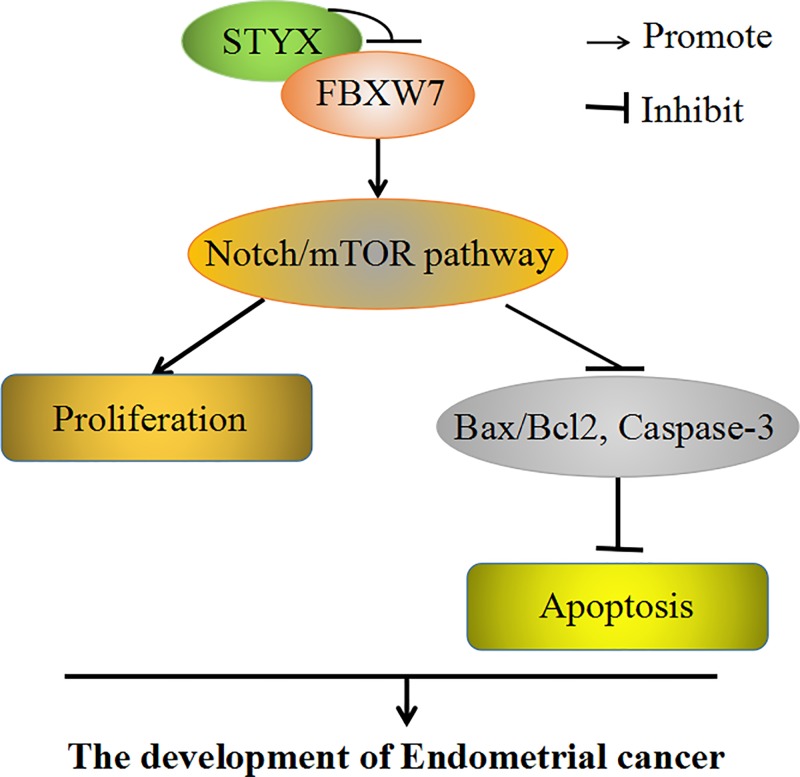
The model that FBXW7 participates the process of EC

Our results also indicated that silencing STYX inhibited cell proliferation of EC cells, whereas silencing FBXW7 partly rebounded the influence of shSTYX on cell proliferation (Figure 6A). Compared with control group, depletion of STYX facilitated apoptosis of EC cells, depletion of STYX and FBXW7 at the same time regained cell apoptosis back to control group cells (Figure 6B). Over-expressing STYX promoted cell proliferation and suppressed apoptosis of EC cells, but DAPT (Notch inhibitor) partially restored this effect of pcDNA3.1-STYX on cell proliferation and apoptosis back to control group cells (Figure 6C,D). Total results testified that STYX adjusted cell proliferation and apoptosis in EC by regulating FBXW7 via Notch–mTOR signaling pathway (Figure 7).

## Discussion

Abnormally expressed FBXW7 has been proved in a lot of tumors, for instance, gastric cancer and colorectal cancer [19,31,32]. Iwatsuki et al. suggested that the expression of FBXW7 in mRNA and protein levels were down-regulated in colorectal cancer [22]. Levine et al. integrate genomic characterization of endometrial cancer, and indicate that FBXW7 has a high frequent mutation in EC [1]. And our results also identified that FBXW7 was low expressed in EC tissues as other various tumors. There’s a study testified that low FBXW7 expression levels in cancers lead to malignant potential, for example tumor size, lymph node metastasis and poor prognosis [33,34]. And FBXW7 acts as a general tumor suppressor in many cancers [16,35,36]. Zhou et al. has identified that FBXW7 inhibits cell proliferation in cervical cancer [37]. Xiang et al. also attests that down-regulated FBXW7 promotes cell proliferation in lung cancer [38]. In the present study, FBXW7 controls the proliferation of EC cells, which is consistent with the study of Zhou and Xiang. A study shows that FBXW7 promotes cell apoptosis in hepatocellular carcinoma [39]. Xia et al. indicated that knockdown of FBXW7 facilitated the ability of proliferation and decreased apoptosis in breast cancer cells [40]. Our studies also exhibited similar results that over-expressing FBXW7 facilitated cell apoptosis in EC. As far as we know, our study was the first to explore the role of FBXW7 in EC.

Recently reports have conformably agreed that STYX plays oncogenic roles in tumors [13,15]. A proof demonstrated that STYX suppresses FBXW7 expression via direct protein–protein interaction in breast cancer cells [41]. In the present study, STYX interacted with FBXW7 was also identified. He et al. suggested that over-expressing STYX promotes CRC cell proliferation [11]. Reiterer et al. certified that STYX knockdown accelerates cell apoptosis in breast cancer cells [41]. And we also showed that silencing STYX decreased cell proliferation and facilitated cell apoptosis in EC. Several studies has attested that silencing FBXW7 counteracts the decreased effect on cell proliferation and promoted effect on cell apoptosis after silencing STYX [11,41]. Likewise, we acquired identical consequence that the function of FBXW7 on cell proliferation and apoptosis was regulated by STYX in EC.

The Notch and mTOR signaling pathway are involved in the carcinogenesis of numerous cancers, and blockade of Notch and mTOR pathway appears to influence cell proliferation in various types of cancers [42–44]. Recently studies have suggested that aberrant activation of the Notch signaling accelerates cell proliferation in endometrial cancer, and suppression of mTOR signaling pathway in EC inhibits tumor initiation and progression [45,46]. Mori et al. demonstrated that FBXW7 modulates cell apoptosis via inhibiting Notch signaling pathway [47]. Our results also showed that over-expressing FBXW7 suppressed Notch signaling related protein and p-mTOR/mTOR, and regulated the proliferation and apoptosis of EC cells through Notch/mTOR signaling pathway.

In brief, this is the first research to attest the consequence of FBXW7 in EC by examining FBXW7 expression, its impact on the development of EC and the latent mechanism. STYX direct interacted with FBXW7 and adjusted FBXW7 expression in EC cells. Over-expression of FBXW7 facilitated the prolifration and suppressed the apoptosis of EC cells, and this course was controlled by STYX. NICD and Hes-1 showed a lower expression after over-expression of FBXW7. The use of Jagged-1 and MHY1485 reversed the function of over-expression of FBXW7 in the aspect of cell proliferation and apoptosis. DAPT also reversed the function of over-expression of STYX in the aspect of cell proliferation and apoptosis. Generally speaking, all of our results demonstrated that STYX/FBXW7 axis participates in the development of endometrial cancer cell via Notch–mTOR signaling pathway.

## Abbreviations

EC: endometrial cancer
FBXW7: F-box and WD40 domain protein 7
PTP: protein tyrosine phosphatase
STYX: serine/threonine/tyrosine interacting protein
